# Cross-linked xenogenic collagen implantation in the sheep model for vaginal surgery

**DOI:** 10.1007/s10397-015-0883-7

**Published:** 2015-02-05

**Authors:** Masayuki Endo, Iva Urbankova, Jaromir Vlacil, Siddarth Sengupta, Thomas Deprest, Bernd Klosterhalfen, Andrew Feola, Jan Deprest

**Affiliations:** 1 Centre for Surgical Technologies, Faculty of Medicine, KU Leuven, Herestraat 49, 3000 Leuven, Belgium; 2 Department of Development and Regeneration, Organ Systems Cluster, Faculty of Medicine, KU Leuven, Herestraat 49, 3000 Leuven, Belgium; 3 Pelvic Floor Unit, University Hospitals KU Leuven, Leuven, Belgium; 4 Institute for Care of Mother and Child, Prague, Czech Republic; 5 Institute for Pathology, Düren Hospital, Düren, Germany

**Keywords:** Graft-related complication, Biological graft, Prolapse, Biomechanics, Contractility

## Abstract

The properties of meshes used in reconstructive surgery affect the host response and biomechanical characteristics of the grafted tissue. Whereas durable synthetics induce a chronic inflammation, biological grafts are usually considered as more biocompatible. The location of implantation is another determinant of the host response: the vagina is a different environment with specific function and anatomy. Herein, we evaluated a cross-linked acellular collagen matrix (ACM), pretreated by the anti-calcification procedure ADAPT® in a sheep model for vaginal surgery. Ten sheep were implanted with a cross-linked ACM, and six controls were implanted with a polypropylene (PP; 56 g/m^2^) control. One implant was inserted in the lower rectovaginal septum, and one was used for abdominal wall defect reconstruction. Grafts were removed after 180 days; all graft-related complications were recorded, and explants underwent bi-axial tensiometry and contractility testing. Half of ACM-implanted animals had palpable induration in the vaginal implantation area, two of these also on the abdominal implant. One animal had a vaginal exposure. Vaginal ACMs were 63 % less stiff compared to abdominal ACM explants (*p* = 0.01) but comparable to vaginal PP explants. Seven anterior vaginal ACM explants showed areas of graft degradation on histology. There was no overall difference in vaginal contractility. Considering histologic degradation in the anterior vaginal implant as representative for the host, posterior ACM explants of animals with degradation had a 60 % reduced contractility as compared to PP (*p* = 0.048). Three abdominal implants showed histologic degradation; those were more compliant than non-degraded implants. Vaginal implantation with ACM was associated with graft-related complications (GRCs) and biomechanical properties comparable to PP. Partially degraded ACM had a decreased vaginal contractility.

## Introduction

Pelvic organ prolapse (POP) develops in half of parous women over 50 years, half of them being symptomatic with only 20 % of them seeking medical help [1, 2]. Lifetime risk of POP surgery is 19 % [3], and up to 25 % require later re-operation [4]. It was suggested that this may be reduced by using synthetic or biological implants [4, 5]. Although durable synthetic meshes are known to achieve good anatomical and functional results for cystocele repair, they may cause graft-related complications (GRCs) in over 10 % of women [4]. Alternative grafts that may reduce the number of GRC yet still provide durable results could be contemplated [6–10].

Biological grafts are derived from either human (autograft or allografts) or animal material (xenografts). Autografts, such as fascia lata, inherently have donor site-related morbidity and also have unpredictable durability [11, 12]. Allografts are retrieved from cadaveric tissue, and although fastidious steps are taken in preparation, concerns regarding transmission of possible prion disease or viruses remained. Xenografts are acellular collagen matrices (ACMs) that are either derived from the dermis, pericardium or small intestinal submucosa of animals that are purposely bred in strictly controlled conditions. Most ACMs are of bovine or porcine origin, which, during their production, undergo various chemicalprocedures (cross-linking, sterilization). After implantation, ACMs are remodelled and/or replaced by connective tissue within variable time periods. The latter can be modified using, e.g. cross-linking agents which leads to the formation of excessive intramolecular and intermolecular chemical bonds preventing decomposition by endogenous collagenases [13]. This alters the properties of the ACM either physically or chemically. A commonly used cross-linking agent is glutaraldehyde (GAD) [13] resulting in durable grafts that are slowly integrated and remodelled. However, residual GAD is cytotoxic and may cause calcification. To prevent this, Neethling et al. developed a multi-step anti-mineralization process called ADAPT® [14]. This enhances crosslink stability, removes residual GAD and modifies the non-bifunctionally reacted GAD residues. The process reduces lipid content and restores tissue elasticity [14]. ADAPT®-treated bovine pericardial patches have been successfully used in surgery of congenital heart defects without demonstrable calcification in a 36-month follow-up period [15]. These promising results demonstrating long-term stability in challenging circumstances are worthwhile considering for translation in pelvic floor surgery. Herein, we used ADAPT®-treated xenografts in a sheep model for transvaginal surgery, studying both the occurrence of GRCs and active and passive biomechanical properties of the vaginal wall. As a reference, we compared outcomes to repairs with light weight polypropylene (PP) implants.

## Materials and methods

### Implants and surgery

This study compares outcomes following vaginal and abdominal mesh insertion of either a xenogenic or synthetic implant in a sheep model. The xenogenic graft was a non-perforated acellular collagen matrix (ACM) derived from bovine pericardium which was cross-linked with an ultra-low concentration (0.05 %) of monomeric GAD. Further preimplantation processing included the so-called ADAPT® anti-calcification procedure and sterilization with propylene oxide [14, 16] (material donated by Prof WML Neethling, Fremantle, Australia). As a control, we used a commercially available monofilament polypropylene (PP) mesh used for vaginal prolapse repair (PP) (Avaulta Solo; 56 g/m^2^, Bard Medical, Covington, GA, USA). The latter was purchased and delivered sterile via the hospital pharmacy. Observations of these animals were earlier reported on elsewhere [17].

**Fig. 1:**
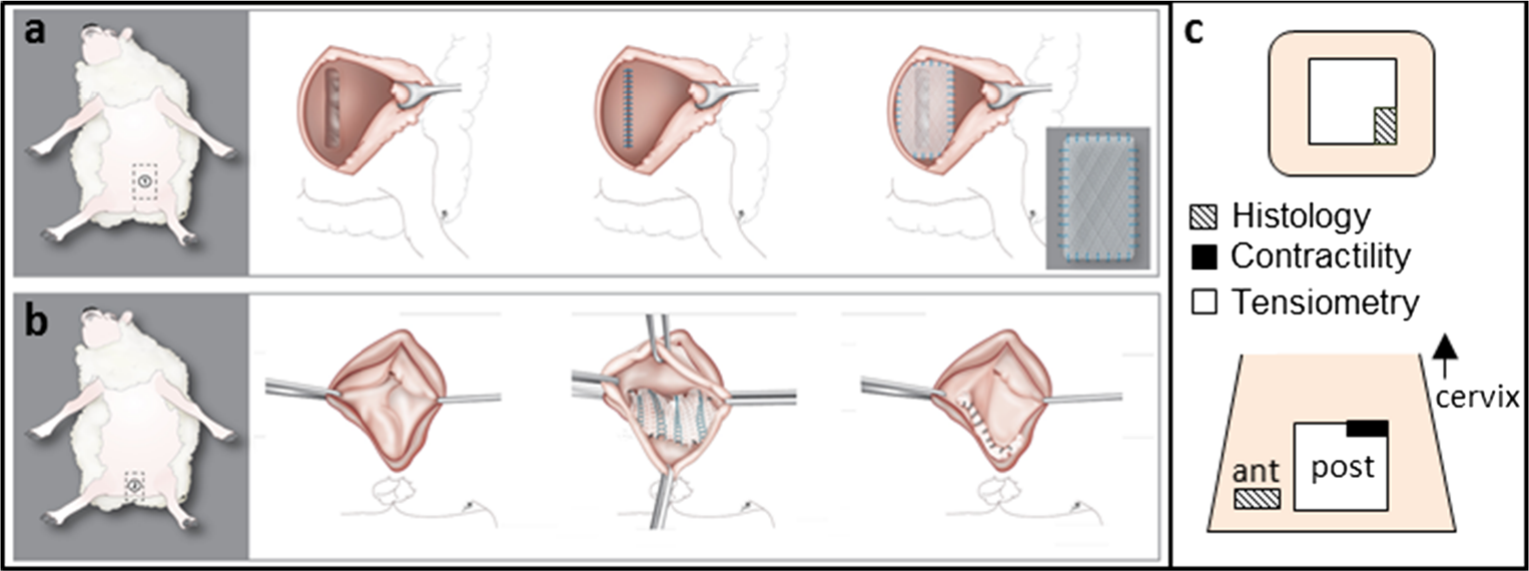
Schematic drawing of abdominal (**a**) and vaginal (**b**) implantation in the sheep model. Specimens explanted (**c**) from the abdomen and anterior (*ant*) and posterior (*post*) vaginal wall were divided according with their respective testing method. The *arrow* is pointing cranially in the direction to the uterine cervix (illustration by Myrthe Boymans)

The anaesthetic, surgical technique and methodology used for outcome evaluation have been described in detail previously [18, 19]. Briefly, 16 parous Texel sheep (mean weight 68 ± 3.5 kg) were obtained from the Zootechnical Centre of the KU Leuven. They underwent simultaneous vaginal and abdominal implantation with either ACM (*N* = 10) or PP (*N* = 6). Surgery was conducted in sterile conditions under general anaesthesia with prophylactic antibiotics at induction and 3 days of postoperative analgesia. Following aqua dissection, a single incision was made in the recto-vaginal septum that was then dissected to create a suitable space for a 35 × 35 mm suture fixed prosthesis (posterior implant) (Fig. 1). Additionally, a 10 × 20 mm graft was inserted in the anterior vaginal wall (anterior implant). Finally, a 50 mm longitudinal paramedian cutaneous incision was made in the anterior abdominal wall, and a 40-mm primarily suture repaired full-thickness fascial incision was overlaid with the same graft as used vaginally (Fig. 1). All implants were fixed with interrupted PP 4/0 prolene sutures (Ethicon). Postoperatively, animals were allowed to move, drink and eat ad libitum and were clinically followed by a veterinarian.

### Outcome measures

On average, 180 days later, animals were euthanized, and during necropsy graft-related complications (GRCs) or the presence of herniation was noted at each of the three implantation sites. Thereafter, the original implant together with the adjacent and ingrown tissue (further referred to as explant) was removed “en bloc” and its dimensions and thickness were determined as an average of three random measurements with a digital micrometer (Mitutoyo, Kawasaki, Japan; accuracy 0.01 mm). Contraction of the explant was defined as the explant area over initial graft area (1225 mm^2^ for vaginal, 2500 mm^2^ for abdominal implant, respectively). Explants were then divided to obtain specimens for histology and biomechanical testing as shown in Fig. 1. For vaginal explants, the anterior specimens were used for histology while the posterior explants were used for active and passive biomechanical testing. The larger abdominal explants were divided for both histology and passive biomechanics.

Histology quantified the inflammatory response and connective tissue formation on 5-μm-thick sections, stained with hematoxylin and eosin (H & E) and Movat pentachrome, using an ordinal scoring system [20, 21]. Two operators blinded to the initial treatment counted foreign body giant cell (FBGC), polymorphonuclear (PMN) cells, newly formed vessels and collagen organization, composition and amount in five randomly chosen areas at the implant–host tissue interface at a magnification × 400 (Zeiss Axioplan 400, Oberkochen, Germany). Infection was classified as either low grade (≥15 PMN per HPF and no clinical evidence of infection) or high grade (presence of micro-abscesses, dense inflammatory infiltrate, fibrin exudation, bleeding and necrosis as well as clinical signs of infection, if any) [22]. All histological scores (FBGCs, PMN, vessel and collagen scores) were at least done in duplicate and averaged.

Passive biomechanics were tested using bi-axial tensiometry by a plunger test on a 500-N Zwick tensiometer with a 200-N cell load (Zwick GmbH & Co. KG, Ulm, Germany) using a protocol defined earlier [18]. A spherical 11.5 mm plunger was passed through an aperture ∅20 mm that exposing the explant compressed in ∅30-mm rings. All explants were placed with the graft facing upward. Records of the force–elongation relationship allowed to define the stiffness (N/mm) of the tested material, which we measured in the low-stress area also called as the “comfort” zone, the average slope of the force–elongation curve and the length of the former zone. These values were determined by TestXpert II software (Zwick GmbH & Co). Active biomechanics of a circumferential-oriented posterior vaginal explant as well as control strip (10 × 8 mm) were assessed by a contractility assay (Fig. 1) [23]. This is an ex vivo assessment quantifying the ability of the smooth muscles present in vaginal tissue strips to contract by immersing them in an organ bath at 80 mM KCl concentration (mN/mm^3^), suggested as a proxy for vaginal function.

### Statistical analysis and ethics committee approval

Data are reported as mean and standard deviation or as median and interquartile range depending on the distribution. Either a Student’s *t* test or a Mann–Whitney *U* test was performed to compare PP and ACM. Chi-square was used for categorical data. Pairs of abdominal and vaginal explants from the same animal were also tested using a paired *t* test or Wilcoxon signed-rank test. All analyses were performed with Prism 5 (GraphPad Software, Inc., La Jolla, CA, USA), and the significance level was set up to *p* < 0.05. Animals were housed in controlled conditions and treated in accordance with current national guidelines on animal welfare. The experiment was approved by the Ethics Committee for Animal Experimentation of the Faculty of Medicine of the KU Leuven.

## Results

### Vaginal versus abdominal implantation of ACM

**Fig. 2:**
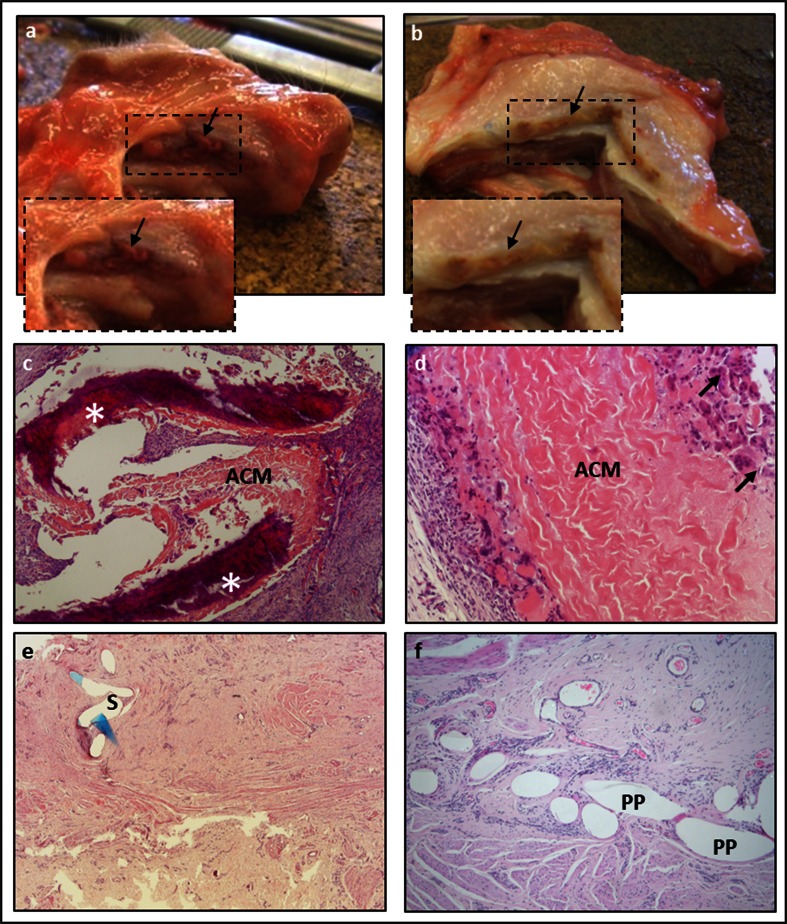
Gross anatomy. Explants from the posterior vaginal wall (**a**) and the abdomen (**b**) with induration and folding visible in the below placed selections. The *dark area* (*arrows*) below the vaginal epithelium and above the abdominal muscles is the location of the implant, which looks complete. In these cases, the material was hard on palpation and surrounded with excessive amount of connective tissue. Histology showed variable host response (**c**–**e**), gradual degradation of ACM with mineral precipitation or calcification (*asterisk*; **c**) and FBGC (*arrow*) on the graft–tissue interface (**d**), or complete ACM degradation where just sutures (*S*) were identifiable (**e**). Polypropylene implant (*PP*) showed uniform response (**f**). (H & E, ×40, ×200, ×25, ×100, respectively)

Five out of ten sheep implanted with ACM developed GRCs at the vaginal implantation site (50 %; Table 1). There was one exposure, two implants showed clinically obvious folding (Fig. 2), and two showed remarkable induration. The total GRC rate for xenogenic implants in the abdominal wall was 30 %. Animals that had induration in their vaginal implants also showed induration of their abdominal implants.

**Table 1:** Paired comparison of outcomes of vaginally and abdominally implanted ACMs

	ACM		Paired comparison
	Abdomen	Posterior vagina	
Graft-related complication	3/10 (30 %)	5/10 (50 %)	ns
Exposure	0/10 (0 %)	1/10 (10 %)	
Folding	0/10 (0 %)	2/10 (20 %)	
Induration	3/10 (30 %)	2/10 (20 %)	
Other gross anatomical findings			
Thickness (mm)	8.22 ± 3.90	6.78 ± 2.27	ns
Material not identifiable	0/10 (0 %)	1/10 (10 %)	ns
Contraction of identifiable mesh	−20.28 % ± 18.24	−61.18 % ± 17.25	0.0008
Biomechanics			
All ewes			
Comfort zone stiffness (N/mm)	0.68 ± 0.2	0.41 ± 0.46	ns
Comfort zone length (mm)	7.18 ± 2.00	8.52 ± 3.22	ns
Exclusion of outlier (*n* = 9)			
Comfort zone stiffness (N/mm) (*n* = 9)	0.73 ± 0.29	0.27 ± 0.19	0.0101
Comfort zone length (mm) (*n* = 9)	7.30 ± 2.00	8.90 ± 3.17	ns

Biomechanical findings are displayed for all animals as well as results without the outlier, and contraction is displayed without those with unidentifiable or extruded material.

**Fig. 3:**
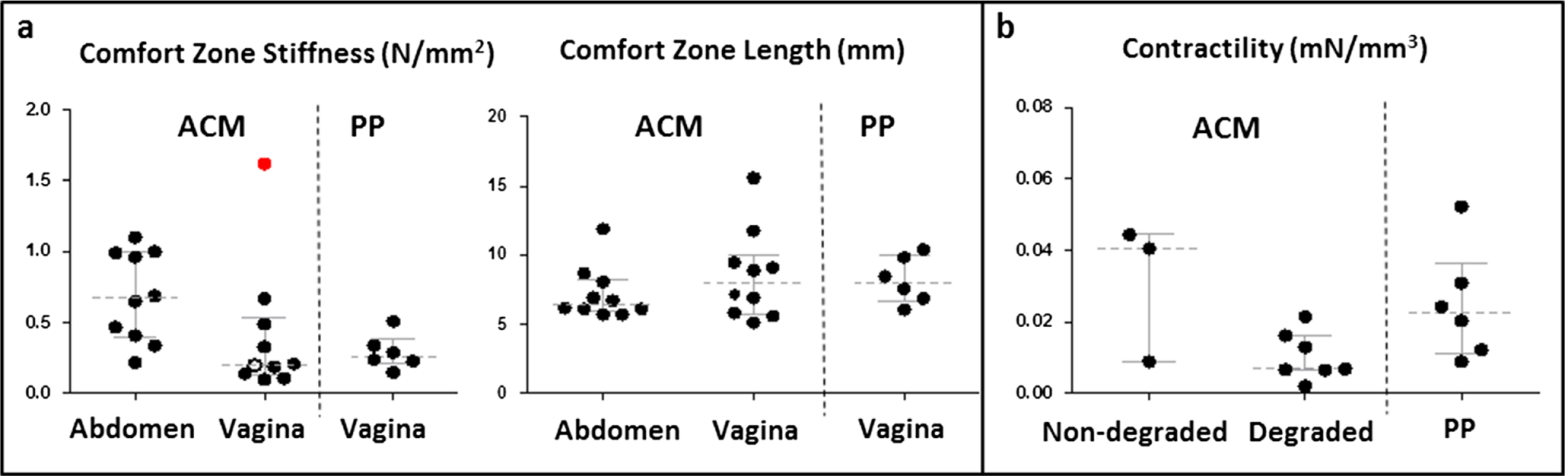
Box plots and individual data of passive (**a**) and active (**b**) biomechanical tests. Individual data are plotted (*black circle*) with median and interquartile range marked with *gray lines*. The not identifiable implant is marked with an *empty circle* and outlier *red circle*

In one vaginal explant, there was macroscopically no visible implant in between the fixation sutures any more. Vaginal and abdominal explants were of comparable thickness, though the contraction rate was almost three times higher in vaginal explants (*p* = 0.0008). There were significant differences between implantation sites for passive biomechanics: vaginal explants were 63 % less stiff than their abdominal counterparts (*p* = 0.01), though the length of the comfort zone was comparable (Fig. 3a). Explants were also categorized by histologic signs of degradation. In the presence of histologic loss of implant integrity, degraded abdominal implants were more compliant than those without degradation (0.44 vs. 0.79 N/mm). Because of the low number, no statistics were done. For vaginal implants, the histology was taken from the anterior side. Of those animals with histologic signs of degradation, their corresponding posterior implants were compared to those without degradation. The compliance of posterior implants of animals with degradation in the anterior implant was lower than in those without (0.33 vs. 0.15 N/mm). Again, no statistics were done.

Histology of vaginal explants was scored on smaller anterior vaginal implants (Table 2). One must remember that this is a different location than the rectovaginal area described above. ACM abdominal explants had higher scores for FBGC (*p* = 0.0078) yet similar amounts of PMN and vascularization as vaginal ACM explants. Scores for collagen organization and composition were similar for both sites, but the amount of collagen was higher in the abdominal ACM explants (*p* = 0.0201). All the above scores were averages. At closer look, explants fall apart in two categories. In 70 % of the anterior vaginal and 30 % of the abdominal explants, we could not trace the typically dense structure of the ACM on histology (Fig. 2d, e). In these cases, there was no measurable inflammation but limited areas of organized and mature connective tissue. Isolated pockets of inflammation were visible only when a suture was accidentally caught in the section. In the other cases, the ACM could be identified. In that case, there was an abundance of inflammatory cells, both FBGC and PMN, as evidenced by their score, as well as connective tissue deposition. In five ACM sections, we observed a precipitated material compatible with calcification (Fig. 2c). Four were abdominal explants; three of them were macroscopically categorized as indurated. In one of these ewes, also the anterior and posterior vaginal explants were indurated. The anterior explant showed calcification on histology, which coincided with histologic signs of infection).

**Table 2:** Histological scores of host response and connective tissue formation following insertion of ACM and PP in the vagina and abdominal wall

Histology	Abdomen	Anterior vagina	Pair comparison	Anterior vagina	Unpaired comparison
	ACM			PP	
	*N = 10*	*N = 10*		*N* = 6	
FBGC	0.8 (0.88)	0.1 (0.53)	0.0078	0.40 (0.25)	
PMN	0.15 (0.38)	0.2 (0.67)		0.05 (0.23)	
Vascularity	1.0 (0.4)	1.15 (0.3)		2.05 (0.55)	0.0023
Collagen organization	1.5 (1.4)	1.4 (0.35)		1.3 (0.55)	
Collagen composition	1.8 (0.4)	2.1 (0.4)		2.3 (0.55)	
Collagen amount	2.7 (0.2)	2.5 (0.2)	0.0201	1.9 (0.35)	0.0061
Calcification	4/10	1/10		0/6	
Scores at the interface with identifiable material on histology	*N = 7*	*N = 3*		*N = 6*	
FBGC	1.1 (0.7)	0.8 (0.35)		0.40 (0.25)	
PMN	0.1 (0.45)	1.8 (0.85)		0.05 (0.23)	
Vascularity	0.7 (0.45)	1.5 (0.45)		2.05 (0.55)	
Collagen organization	1.4 (1.0)	0.8 (0.5)		1.3 (0.55)	
Collagen composition	1.8 (0.3)	2.4 (0.3)		2.3 (0.55)	
Collagen amount	2.8 (0.3)	2.8 (0.1)		1.9 (0.35)	

Scores are displayed for all explanted specimens and also for those with identifiable ACM. For later, statistics was not carried out due to a few specimens. Data for vaginal PP implants were previously published by Feola et al. 2014 [17]

*FBGC* foreign body giant cell, *PMN* polymorphonuclears

### Vaginal contractility and comparison to PP vaginal implants

Figure 3 displays the vaginal contractility findings. Posterior ACM explants where no graft was recognizable on histology (*n* = 7) and the corresponding anterior vaginal implant had a 68 % lower contractility than those with recognizable material (*n* = 3; Fig. 3b). Again, in view of the very low numbers, no statistics were attempted.

We compared the vaginal contractility and other findings to observations made earlier when using a PP vaginal implant (Table 3). In the PP group, there was one case of GRC (folding), yet this apparently lower number was not significant. However, the 17 % contraction rate of PP explants was significantly less than for ACM (*p* = 0.0009). Passive biomechanics of all vaginal ACM were comparable to observations in the PP explants. Compliance of degraded ACM (0.33 N/mm) was within the range of what was measured in PP (0.29 N/mm) explants. Active biomechanical findings were also comparable. However, the values of three animals with recognizable ACM were in the range of the PP-implanted animals. Conversely, the forces generated by the seven animals without degradation in the anterior ACM were 60 % lower than those of PP explants (*p* = 0.048) (Fig. 3b).

**Table 3:** Outcomes and test results of vaginally implanted ACM and PP and their unpaired comparison (significance level *p* < 0.05)

	ACM	PP	Unpaired comparison
	Posterior vagina	Posterior vagina	
	*N* = 10	*N* = 6	
Graft-related complication	5/10 (50 %)	1/6 (16.6 %)	0.0367
Exposure	1/10 (10 %)	0/6 (0 %)	
Folding	2/10 (20 %)	1/6 (16.6 %)	
Induration	2/10 (20 %)	0/6 (0 %)	
Contraction of identifiable mesh	−53.82 % ± 10.20 (*n* = 7)	−23.06 % ± 16.63	0.0009
Biomechanics			
Comfort zone stiffness (N/mm)	0.27 ± 0.19 (*n* = 9)	0.29 ± 0.12	ns
Comfort zone length (mm)	8.89 ± 3.17 (*n* = 9)	8.18 ± 1.67	ns
Contractility (mN/mm^3^)	0.017 ± 0.015 (*n* = 10)	0.025 ± 0.016	ns
Degraded (*n* = 7)	0.010 ± 0.007	0.025 ± 0.016	0.0481
Non-degraded (*n* = 3)	0.031 ± 0.019	–	–

The histology of vaginal explants with PP differed completely from those with recognizable ACM (Fig. 2f). PP induced a mild inflammation, with few cells, nearly all macrophages or FBGC, and less collagen deposition. These specimens showed no histologic signs of infection.

## Discussion

In this study, we used the ovine model to document outcomes following insertion of a cross-linked ACM in the rectovaginal septum and, for histology, insertion of a smaller piece into the anterior vaginal wall. We used an investigational product that was treated with the ADAPT® procedure to prevent calcification, reduce lipid content and restore tissue flexibility. We used a double control, i.e. an internal control by implantation of the same material at a control site (the abdominal wall) and an external control by implantation of a durable PP implant at the same (vaginal) site.

Conceptually, ACM is the ideal matrix for gradual integration into the host. In order to avoid adverse reactions, ACMs are made free of allergens, DNA and other pathogens. Cross-linking should make them resistant to endogenous collagenase activity. However, several experimental and clinical studies report unfavourable outcomes with the use of a variety of ACM. Clinically, both GRC and failure were reported [24–27]. Also, our experiment revealed a number of GRC. The first striking observation was the presence of calcification on histology. This happened at both anatomical locations and not in the control group. Calcification of GA cross-linked grafts has been tied to residual non-viable cells and cellular debris that were not removed during GAD pretreatment. They serve as nucleation sites for calcium phosphate minerals. GAD modifies phosphorous-rich cell membranes that are capable of mineralization using calcium present in the extracellular fluid [28, 29]. This adverse effect was meant to be prevented by the ADAPT® procedure, which has been earlier shown to work in previous experimental studies in rats and sheep [30–32]. Clinical studies on the use of the same material for cardiac defects showed minimal or no mineral precipitation in the matrices [15]. This was reassuring because pathological calcification is a feared complication in cardiac surgery, certainly in young patients [33, 34]. Another identified risk factor for calcification of grafts is the occurrence of infection or folding [35, 36]. These may add to other surgical factors, such as haemorrhage, or damage by surgical handling. To our knowledge, no previous studies have looked at the use of this material for surgical repair of the abdominal wall or a vaginal environment.

The most common local observation was palpable induration at the implant site. Histologically, this did not always coincide with calcification but may also with contraction, folding and wrinkling. There are no published data on contraction rates following insertion of cross-linked ACM in the vagina. Studies on abdominal insertion showed various results. Ozog et al. reported minimal or unchanged dimensions in 180 days with Pelvicol in rats, however, without visible deformation [37]. Conversely, Jenkins et al. reported a 50 % area reduction and wrinkling for abdominal wall reconstruction after 3 months in mini pigs using Collamend (another cross-linked porcine dermis; Bard, Davol, RI) [38]. They explained the process by the presence of encapsulation, though we observed that process also in non-contracted ACM [37]. Contraction is, however, not the privilege of ACM, as significant contraction rates have been reported following PP implantation both in the vagina (45 %) and abdomen (10 %) 90 days after surgery [18].

We also observed various stages of graft degradation in 30 % of abdominal and 70 % of vaginal explants. The occurrence of degradation of cross-linked ACM has been reported by several groups in different models [39–42]. Claerhout et al. documented degradation in rabbit abdominal Pelvicol (Bard, Haasrode, Belgium) implants from 180 days onwards [40], and Pierce documented the same for vaginal Pelvisoft implants [41]. In a clinical study, we saw the same when Pelvicol was used for laparoscopic sacrocolpopexy, leading to recurrence [24]. Graft degradation coincided in 8 out of 9 cases with an abundance of foreign body (giant) cells on biopsy [43]. Description of the exact time course of degradation is currently impossible since clinical, as experimental studies usually involve only one time point. Interestingly, degradation does not occur in the same way at both locations. Pierce et al. showed more degradation of Pelvisoft (Bard, Covington, GA) 270 days following vaginal than abdominal implantation [44]. The same difference was present in our study. It is not possible to determine whether this is a faster or more vigorous process. However, Abramov et al. showed differences between the wound healing response in the vagina and in the abdomen, which may lead to another remodelling process [45].

Vaginal ACM explants were around 2.7 times less stiff than their abdominal counterparts. To our knowledge, there are no studies on biomechanics following simultaneous ACM implantation in both locations, so we do not have references to compare to. However, differences in biomechanics for these two environments were studied following insertion of PP (Gynemesh; Ethicon, Somerville, NJ) in a rabbit [44]. Vaginal explants were 1.5 less stiff than abdominal explants. Both observations follow a trend reported on biomechanics of cadaverous native tissue from the vagina and abdominal aponeurosis [46]. Gabriel et al. reported the vaginal wall being four times less stiff than abdominal aponeurosis. There are, to our knowledge, no published reference data on the biomechanics of native vaginal tissue and abdominal wall.

Graft degradation is another factor of relevance, as it interferes with the biomechanical properties. For that reason, we compared stiffness of those animals where no ACM was visible anymore versus those where the material was conserved. Effects seemed to be different according to the location of implantation. Yet, low numbers preclude actual statistical assessment, and more importantly, degradation was determined on the anterior implant, whereas biomechanics on the posterior implant. For those reasons, we prefer not to make any firm conclusions on this matter.

Smooth muscle contractility showed, at the first glance, no difference for the explants using different implant materials. Further analysis into two subgroups revealed a 68 % decline of forces in group without visible ACM. When there was still detectable ACM, findings were comparable to PP-implanted tissues. In other words, the absence of material coincides with reduction in contractility. In line with other studies, we further demonstrated that ACM implants affect smooth muscle contractility [47]. This is somewhat counter-intuitive unless one assumes that earlier in time, the implant may have already compromised contractility, e.g. by a process of stress shielding, as described by others [47–49]. To elucidate that, one would, however, need a study with several time points and appropriate native tissue controls, and the mechanisms remain therefore unexplained.

Our study has several limitations. Again, we only studied one time point, precluding insight into the time course of the host response; hence, it cannot learn on the processes driving the adverse events neither. Another is the use of a semiquantitative histological scoring system and limited staining methods. Though they are descriptive, it would be much more informative to also have biochemical and molecular read outs to document details on the nature of the immune response, neovascularization, collagen metabolism and nerve ingrowth [50]. This might be difficult to do, as those tools are not readily available in sheep and also add significantly to the cost. Further, we had to resort to use a second vaginal location for implantation so that we could obtain sufficient tissue for histology, without compromising the availability of tissue for biomechanical testing. Further, there are the limitations of the model; even though sheep can be used as a model for vaginal surgery as well as prolapse [18], it remains mostly an asymptomatic quadruped, with clinically different anatomical and pelvic floor loads, hence not an ideal disease model. It has however several advantages: apparently, sheep may clinically develop obvious pelvic floor relaxation and some degree of prolapse after delivery; further, this is a widely available, affordable alternative to the primate, which would be the closest model that we could think off.

In conclusion, we demonstrated that despite treatment with ADAPT®, ACM insertion into the rectovaginal septum was associated with a number of local adverse effects. Apart from that, the passive biomechanical properties were not better than what was obtained when a PP mesh was used. Following degradation of the ACM, there was a decrease in smooth muscle contractility. The ideal implant material has apparently not yet been identified.
